# *DNMT3B* rs1569686 and rs2424913 gene polymorphisms are associated with positive family history of preterm birth and smoking status 

**DOI:** 10.3325/cmj.2020.61.8

**Published:** 2020-02

**Authors:** Anita Barišić, Maja Kolak, Ana Peterlin, Nataša Tul, Milena Gašparović Krpina, Saša Ostojić, Borut Peterlin, Nina Pereza

**Affiliations:** 1 Department of Medical Biology and Genetics, Faculty of Medicine, University of Rijeka, Rijeka, Croatia; 2 Department of Oncology, Clinical Hospital Center Rijeka, Rijeka, Croatia; 3 Clinical Institute of Medical Genetics, University Medical Center Ljubljana, Ljubljana, Slovenia; 4 Department of Obstetrics and Gynecology, University Medical Center Ljubljana, Ljubljana, Slovenia; 5 Department of Obstetrics and Gynecology, Clinical Hospital Center Rijeka, Rijeka, Croatia; 6 Division of Obstetrics and Gynecology, Clinical Institute of Medical Genetics, University Medical Center Ljubljana, Ljubljana, Slovenia; 7 Department of Biology, Faculty of Medicine, University of Rijeka, Rijeka, Croatia

## Abstract

**Aim:**

To evaluate the association between spontaneous preterm birth (SPTB) and DNA methyltransferase (*DNMT*)1, 3A, 3B, and 3L gene polymorphisms, and their contribution to the clinical characteristics of women with SPTB and their newborns.

**Methods:**

This case-control study, conducted in 2018, enrolled 162 women with SPTB and 162 women with term delivery. *DNMT1* rs2228611, *DNMT3A* rs1550117, *DNMT3B* rs1569686, *DNMT3B* rs2424913, and *DNMT3L* rs2070565 single nucleotide polymorphisms were genotyped using polymerase chain reaction and restriction fragment length polymorphism methods. The clinical characteristics included in the analysis were family history of preterm birth, maternal smoking, maternal age, gestational week at delivery, and fetal birth weight.

**Results:**

*DNMT* gene polymorphisms were not significantly associated with SPTB. *DNMT3B* rs1569686 and rs2424913 minor alleles (T) were significantly more frequent in women with familial PTB than in women with non-familial PTB, increasing the odds for familial PTB 3.30 and 3.54 times under dominant genetic models. They were also significantly more frequent in women with SPTB who smoked before pregnancy, reaching the most significant association under additive genetic models (odds ratio 6.86, 95% confidence interval 2.25-20.86, *P* < 0.001; odds ratio 3.77, 95% confidence interval 1.36-10.52, *P* = 0.011, respectively).

**Conclusions:**

*DNMT3B* rs1569686 and rs2424913 gene polymorphisms might be associated with positive family history of PTB and smoking status.

Preterm birth (PTB), defined as birth before the 37th completed week of gestation, is the leading cause of neonatal mortality and morbidity (1). It also significantly increases the risk of long-term health complications compared with term birth (2). Up to 25% of PTBs are medically induced and 50% are initiated spontaneously with intact fetal membranes (SPTB or idiopathic PTB) (3,4). Due to its heterogeneous etiology, SPTB is considered a clinical syndrome (5). A recognized risk factor for SPTB is maternal and/or fetal (epi)genetic predisposition, which has been confirmed in many epidemiological studies (6-8).

DNA methylation patterns guide temporal and tissue-specific gene expression and ensure genome stability. These patterns are extensively modified during gametogenesis and prenatal development (8-10), which makes DNA methylation a good predictor of gestational age at or near birth and a source of information related to the developmental stage (11). Epigenetic alterations were associated with PTB, and global and site-specific DNA methylation patterns were changed in maternal blood, placenta, and cord blood of preterm newborns (12-17). DNA methylation among preterm infants is influenced by both prenatal and postnatal environmental factors, such as maternal stress, social deprivation, and smoking (18-22).

During methylation process, methyl groups are transferred to cytosines by DNA methyltransferases (DNMT), among which *DNMT1*, *DNMT3A*, and *DNMT3B* are the major catalytically active enzymes (23,24). *DNMT1* binds to hemi-methylated DNA and is responsible for the maintenance of established patterns, whereas *DNMT3A* and *DNMT3B* guide *de novo* methylation. Unlike the other DNMTs, *DNMT3L* is an enzymatically inactive regulatory factor that binds to *DNMT3A* and *DNMT3B* and increases their activity (23).

Considering that single nucleotide polymorphisms (SNP) in *DNMT* genes might affect the genes’ expression and consequently methylation, several studies assessed the association of these SNPs with different human reproductive disorders. Polymorphisms of *DNMT1* and *DNMT3A* genes were found to be associated with male infertility and spontaneous abortion after assisted reproduction or natural conception, respectively (25,26). *DNMT3L* gene variants affected birth-weight and were associated with male infertility and ovarian endometriosis (27-29), while maternal *DNMT3B* SNPs increased the risk for PTB and Down syndrome (27,30,31).

The present study examines the potential association between maternal *DNMT1*, *DNMT3A*, *DNMT3B*, and *DNMT3L* gene polymorphisms and SPTB. To identify the factors that cause epigenetic modifications related to SPTB, we also evaluated the association between *DNMT* gene polymorphisms and various clinical characteristics of women with SPTB and their newborns (family history of PTB, maternal smoking before pregnancy, maternal age and gestational week at delivery, and fetal birth weight).

## PATIENTS AND METHODS

### Patients

This case-control study, conducted in 2018, enrolled Slovenian and Croatian women who gave birth at the Division of Perinatology, Department of Obstetrics and Gynecology, University Medical Center in Ljubljana, Slovenia and Department of Obstetrics and Gynecology, Clinical Hospital Centre of Rijeka, Croatia. All participants gave written informed consent. The samples collected in Rijeka are part of the TransMedri Biobank – a bank of biosamples for the investigation of preterm birth (EU-FP7 Regpot-2010-5, Faculty of Medicine, University of Rijeka). The study was approved by the Slovenian National Medical Ethics Committee (98/12/10, 2010) and the Ethics Committee for Biomedical Research of the Faculty of Medicine, University of Rijeka (2170-29-02/15-17-2, 2017).

The patient group included 162 women with SPTB (113 Slovenian and 49 Croatian). Demographic and clinical data of women with SPTB and their newborns were collected in accordance with the guidelines for genetic epidemiology studies on PTB (2) by means of a self-developed interviewer-administered questionnaire. As described in more detail in our previous study (32), all women with SPTB had singleton pregnancies following natural conception and spontaneous initiation of PTB before the 37th week of gestation. Gestational age was estimated from the last menstrual period and confirmed by ultrasound in the first trimester. When the difference between the two estimates exceeded seven days, gestational age was revised according to the ultrasound measurement. The exclusion criteria for patients were the known risk factors for PTB, including diabetes, hypertension, kidney disease, autoimmune conditions, allergic diseases, birth canal infections, *in vitro* fertilization, and pregnancy complications. None of the live-born children had congenital anomalies or evidence of infection. Additional maternal and newborn characteristics are shown in Table 1. The control group enrolled 162 age- and parity-matched women (119 Slovenian and 43 Croatian) who had a term singleton birth after an uncomplicated pregnancy.

**Table 1:** Characteristics of women with spontaneous preterm birth (SPTB) and controls

	No (%) of			
	cases*	controls^†^		P
Maternal characteristics					
Mean age at delivery (years)^‖^	30 (17-44)		30 (20-42)	0.755^‡^	
Gestational age at delivery			37-41		
extremely preterm <28 week	10 (6.4)				
very preterm 32-28 weeks	20 (12.7)				
moderate to late preterm 32-37 weeks	127 (80.9)				
Smoking before pregnancy					
yes	45 (71.3)		23 (20.7)	0.184^§^	
no	112 (28.7)		88 (79.3)		
Smoking during pregnancy					
yes	19 (12.1)		13 (11.7)	0.925^§^	
no	138 (87.9)		98 (88.3)		
Previous PTB					
yes	13 (8.3)		0		
no	144 (91.7)				
Familial PTB					
yes	48 (30.6)		0		
no	109 (69.4)				
**Newborn characteristics**
birth weight (grams)^‖^	2403 (620-3915)		3456 (1570-4560)	<0.001^‡^	
congenital anomalies	0		0		
evidence of infection	0		0		

*epidemiological data were available for 157/162 (97%) women with SPTB.

†epidemiological data were available for 111/162 (69%) controls.

‡t-test.

§χ^2^ test.

‖median and range.

### DNA isolation and genotyping

Genomic DNA was isolated from peripheral blood leukocytes by standard procedure with a commercially available kit (Qiagen FlexiGene DNA kit, Qiagen GmbH, Hilden, Germany) and stored at -20°C.

*DNMT1* rs2228611, *DNMT3A* rs1550117, *DNMT3B* rs1569686, *DNMT3B* rs2424913, and *DNMT3L* rs2070565 SNPs were genotyped using a combination of polymerase chain reaction (PCR) and restriction fragment length polymorphism (RFLP). Primers, PCR and RFLP conditions were modified from the previously published literature (Supplementary material 1) (33-36). Polymerase chain reaction was carried out in thermal cyclers (Mastercycle personal, Eppendorf, Hamburg, Germany and 2720 Thermal Cycler, Applied Biosystems, Carlsbad, CA, USA). All restriction enzymes were obtained from New England Biolabs (Ipswich, MA, USA), and reactions were performed in accordance with the manufacturer’s recommendations. PCR products and restriction fragments were separated using electrophoresis on 3% agarose gels stained with GelRed^TM^ (Olerup SSP^®^, Saltsjöbaden, Sweden).

### Statistical analysis

Normality of distribution was tested with the Kolmogorov-Smirnov test. The Pearson chi square test was used to examine differences in genotype and allele frequencies between various groups of participants. Odds ratios (OR) and 95% confidence intervals (CI) were calculated to determine the association between DNMT gene polymorphisms and SPTB. The *t* test was used for comparison of age and fetal birth weight means between patients and controls, whereas one-way analysis of variance (ANOVA) was used for the comparison of age and fetal birth weight means between the groups with different genotypes of DNMT gene polymorphisms. The level of statistical significance was set at *P* less than 0.05. Statistical analyses were performed with Statistica for Windows, version 13.3 (StatSoft, Inc., Tulsa, OK, USA) and MedCalc for Windows, version 14.12.0. (MedCalc Software, Mariakerke, Belgium). Statistical power was calculated with ClinCalc LLC (*https://clincalc.com/stats/samplesize.aspx*) and Hardy-Weinberg equilibrium was calculated using Simple Hardy-Weinberg Calculator – Court Laboratory (Washington State University College of Veterinary Medicine, Pullman, WA, USA).

## RESULTS

### Genetic association between DNMT gene polymorphisms and SPTB

Cases and controls did not significantly differ in the distribution of genotype or allele frequencies of *DNMT1* rs2228611, *DNMT3A* rs1550117, *DNMT3B* rs1569686, *DNMT3B* rs2424913, and *DNMT3L* rs2070565 SNPs (Table 2). Neither of the polymorphisms was associated with SPTB (data not shown). All genotype frequencies in cases and controls were in Hardy-Weinberg equilibrium (data not shown). The study had 80% power to detect a 2-fold increase in the minor alleles of all SNPs.

**Table 2:** Genotype and allele frequencies of DNA methyltransferase (*DNMT*) gene polymorphisms in women with spontaneous preterm birth (SPTB) and controls

	No (%) of			
	cases	controls	Χ^2^	P
***DNMT1***				
**rs2228611**				
**genotype**				
AA	62 (38.3)	54 (33.3)	1.09	0.581
AG	74 (45.7)	83 (51.2)		
GG	26 (16.0)	25 (15.5)		
**allele**				
A	198 (61.1)	191 (58.9)	0.32	0.575
G	126 (38.9)	133 (41.1)		
***DNMT3A***				
**rs1550117**				
**genotype**				
GG	135 (83.3)	128 (79.0)	1.02	0.601
AG	26 (16.1)	33 (20.4)		
AA	1 (0.6)	1 (0.6)		
**allele**				
G	296 (91.4)	289 (89.2)	0.86	0.353
A	28 (8.6)	35 (10.8)		
***DNMT3B***				
**rs1569686**				
**genotype**				
GG	67 (41.4)	57 (35.2)	2.54	0.281
TG	76 (46.9)	77 (47.5)		
TT	19 (11.7)	28 (17.3)		
**allele**				
G	210 (64.8)	191 (58.9)	2.36	0.124
T	114 (35.2)	133 (41.1)		
**rs2424913**				
**genotype**				
CC	60 (37.0)	48 (29.6)	2.62	0.270
TC	79 (48.8)	83 (51.2)		
TT	23 (14.2)	31 (19.2)		
**allele**				
C	199 (61.4)	179 (55.2)	2.54	0.111
T	125 (38.6)	145 (44.8)		
***DNMT3L***				
**rs2070565**				
**genotype**				
CC	56 (34.5)	50 (30.9)	1.89	0.389
TC	89 (55.0)	87 (53.7)		
TT	17 (10.5)	25 (15.4)		
**allele**				
C	201 (62.0)	187 (57.7)	1.26	0.262
T	123 (38.0)	137 (42.3)		

### Association of DNMT gene polymorphisms with clinical characteristics of women with SPTB and their newborns

Individually, both *DNMT3B* rs1569686 and rs2424913 minor alleles (T) were more frequent in women with familial PTB than in women with non-familial PTB (Χ^2^ = 10.31, *P* = 0.006 and Χ^2^ = 13.96, *P* < 0.001, respectively) (Table 3) and increased the odds for familial PTB 3.30 and 3.54 times under the dominant genetic models (TT + TG vs GG and TT + TC vs CC) (95% CI 1.53-7.14, *P* = 0.003 and 95% CI = 1.56-8.01, *P* = 0.002, respectively) (Table 4).

**Table 3:** Genotype and allele frequencies of *DNMT3B* gene polymorphisms in women with SPTB according to family history of PTB*

	No. (%) of women with			
	non-familial PTB	familial PTB	Χ^2^	P
***DNMT3B***				
**rs1569686**				
**Genotype**				
GG	54 (49.6)	11 (22.9)	10.31	0.006
TG	45 (41.3)	28 (58.3)		
TT	10 (9.1)	9 (18.8)		
**Allele**				
G	153 (70.2)	50 (52.1)	9.55	0.002
T	65 (29.8)	46 (47.9)		
**rs2424913**				
**Genotype**				
CC	49 (45.0)	9 (18.8)	13.96	<0.001
TC	50 (45.9)	26 (54.2)		
TT	10 (9.1)	13 (27.0)		
**Allele**				
C	148 (67.9)	44 (45.8)	13.65	<0.001
T	70 (32.1)	52 (54.2)		

*DNMT – DNA methyltransferase; SPTB – spontaneous preterm birth.

**Table 4:** Association of *DNMT3B* gene polymorphisms with familial PTB*

Genetic models	Familial vs non-familial PTB	
	OR (95% CI)	P
**rs1569686**		
TT vs TG+GG	2.28 (0.86-6.05)	0.096
TT+TG vs GG	3.30 (1.53-7.14)	0.003
TT vs TG	1.45 (0.52-3.99)	0.477
TT vs GG	4.42 (1.46-13.40)	0.009
GG vs TG	0.33 (0.15-0.73)	0.006
T vs G	2.17 (1.32-3.55)	0.002
**rs2424913**		
TT vs TC+CC	3.68 (1.48-9.14)	0.005
TT+TC vs CC	3.54 (1.56-8.01)	0.002
TT vs TC	2.50 (0.96-6.47)	0.059
TT vs CC	7.07 (2.38-21.02)	<0.001
CC vs TC	0.35 (0.15-0.83)	0.017
T vs C	2.49 (1.53-4.09)	<0.001

*OR – odds ratio; CI – confidence interval; DNMT – DNA methyltransferase; PTB – preterm birth.

The individual analysis of *DNMT3B* SNPs showed that rs1569686 and rs2424913 T alleles were also significantly more frequent in patients with SPTB who had smoked than patients who had not smoked before pregnancy (Χ^2^ = 10.12, *P* = 0.001 and Χ^2^ = 5.35, *P* = 0.021, respectively) (Table 5), reaching the most significant association under the additive genetic models (TT vs GG and TT vs CC) (OR 6.86, 95% CI 2.25-20.86, *P* < 0.001 and OR 3.77, 95% CI 1.36-10.52, *P* = 0.011, respectively, Table 6). None of the other polymorphisms contributed to the clinical characteristics of women with SPTB and their newborns (data not shown).

**Table 5:** Genotype and allele frequencies of *DNMT3B* gene polymorphisms according to smoking before pregnancy*

	No. (%) of				No. (%) of			
*DNMT3B*	SPTB non-smokers	SPTB smokers	Χ^2^	P	controls non-smokers	controls smokers	Χ^2^	P
**rs1569686**								
**genotype**								
GG	52 (46.4)	13 (28.9)	13.49	0.001	36 (40.9)	7 (30.4)	0.87	0.647
TG	53 (47.3)	20 (44.4)			40 (45.5)	12 (52.2)		
TT	7 (6.3)	12 (26.7)			12 (13.6)	4 (17.4)		
**allele**								
G	157 (70.1)	46 (51.1)	10.12	0.001	112 (63.6)	26 (56.5)	0.79	0.376
T	67 (29.9)	44 (48.9)			64 (36.4)	20 (43.5)		
**rs2424913**								
**genotype**								
CC	45 (40.2)	13 (28.9)			27 (30.7)	6 (26.1)	0.19	0.911
TC	56 (50.0)	20 (44.4)	7.53	0.023	47 (53.4)	13 (56.5)		
TT	11 (9.8)	12 (26.7)			14 (15.9)	4 (17.4)		
**allele**								
C	146 (65.2)	46 (51.1)	5.35	0.021	101 (57.4)	25 (54.4)	0.14	0.711
T	78 (34.8)	44 (48.9)			75 (42.6)	21 (45.6)		

*DNMT – DNA methyltransferase; SPTB – spontaneous preterm birth.

**Table 6:** Association of *DNMT3B* gene polymorphisms with smoking before pregnancy*

Genetic models	SPTB		Controls	
**OR (95% CI)**	**P**	**OR (95% CI)**	**P**
**rs1569686**				
TT vs TG+GG	5.45 (1.98-14.99)	0.001	1.33 (0.39-4.59)	0.649
TT+TG vs GG	2.13 (1.01-4.49)	0.045	1.58 (0.59-4.24)	0.361
TT vs TG	4.54 (1.57-13.17)	0.005	1.11 (0.30-4.09)	0.874
TT vs GG	6.86 (2.25-20.86)	<0.001	1.71 (0.43-6.89)	0.447
GG vs TG	0.66 (0.29-1.47)	0.311	0.65 (0.23-1.83)	0.412
T vs G	2.24 (1.36-3.71)	0.002	1.35 (0.69-2.60)	0.376
**rs2424913**				
TT vs TC+CC	3.34 (1.35-8.28)	0.009	1.11 (0.33-3.77)	0.864
TT+TC vs CC	1.65 (0.78-3.49)	0.187	1.25 (0.45-3.53)	0.668
TT vs TC	3.05 (1.16-8.01)	0.023	1.03 (0.29-3.68)	0.960
TT vs CC	3.77 (1.36-10.52)	0.011	1.29 (0.31-5.32)	0.729
CC vs TC	0.81 (0.36-1.80)	0.604	0.80 (0.27-2.36)	0.690
T vs C	1.79 (1.08-2.94)	0.022	1.13 (0.59-2.17)	0.711

*OR – odds ratio; CI – confidence interval; DNMT – DNA methyltransferase.

## DISCUSSION

This study indicates that maternal *DNMT3B* rs1569686 and rs2424913 SNPs might be susceptibility factors for SPTB in women who had a positive family history of PTB and had smoked before pregnancy. Although genotype and allele frequencies of *DNMT3B* rs1569686 and rs2424913 SNPs were similar in cases and controls, a subgroup analysis of women with SPTB yielded two significant associations for both polymorphisms. First, the minor (T) allele of rs1569686 or rs2424913 *DNMT3B* polymorphism, in both homozygous and heterozygous form, increased the odds for familial PTB 3.30 and 3.54-fold, respectively, compared with the homozygous form of the major alleles (GG and CC). Positive family history is an independent risk factor and one of the main risk factors for PTB (37-39). Intergenerational influences include both genetic and epigenetic factors, meaning that both the inherited genetic predisposition to PTB and the mother’s lifestyle affect her own and the next generation’s health status (37). *DNMT3B* rs2424913 and rs1569686 are located in the 3′-untranslated and promoter regions of *DNMT3B* gene, 149 and 579 base pairs, respectively, upstream from the transcription start site. The role of rs2424913 SNP is to regulate the expression of *DNMT3B* gene, while the T allele increases promoter activity (38,39) and affects miRNA binding site (40). The functional role of rs1569686 SNP is still controversial, although *in silico* analysis showed that the T allele might affect the binding activity for several transcription factors (40). A previous study reported that both maternal and infant *DNMT3B* rs1569686 and rs2424913 gene polymorphisms influenced inter-individual variation in global DNA methylation (41). In addition, the T alleles of both variants, both in homozygous and heterozygous forms, were associated with the risk of several diseases, mostly different cancer types (42-44). Interestingly, rs1569686 TT genotype and T allele were overrepresented in patients with schizophrenia and positive family history of psychiatric illness (40).

The second important finding in our study was the association of the minor (T) alleles of *DNMT3B* rs1569686 and rs2424913 with maternal smoking, one of the previously confirmed environmental risk factors for SPTB (11,45). Maternal smoking in the pre- and peri-conception period (46,47), as well as throughout pregnancy (45), significantly increased the risk for PTB. For example, Haas et al (47) showed that pre-conception smoking increased the odds for PTB 2-fold (95% CI 1.29-3.75). In our study, women who smoked and were homozygous for *DNMT3B* rs1569686 TT genotype and rs2424913 TT genotype had respectively 6.86-fold and 3.77-fold higher odds for SPTB compared with GG and CC carriers. Interestingly, the lack of significant difference in genotype and allele frequencies between control non-smokers and smokers confirms that smoking before pregnancy combined with TT genotype is an additional risk factor for SPTB. This finding shows that smoking can negatively affect epigenetic modifications in the pre-conception period, especially during ovarian follicular development (48). Previously, maternal smoking has been shown to adversely affect ovarian reserve and oocyte quality (49) and clinical outcomes of assisted reproductive technologies (50), which most likely have epigenetic etiology. As shown by a large epigenome wide association study, smoking changed DNA methylation pattern at multiple genomic loci, which was only partially reversible upon smoking cessation (51). Also, maternal smoking was independently associated with reduced site-specific DNA methylation among preterm infants at birth, both in mothers who quit smoking before pregnancy and those who continued to smoke (52). The spatially and temporally indispensable roles of *de novo* methyltransferase *DNMT3B* during oogenesis and early embryonic development might be affected by the exposure to harmful environmental factors. In humans, *DNMT3B* transcript is present from the primordial follicle stage onwards, but at the germinal vesicle stage its protein is no longer detected in the nucleus, indicating that *de novo* DNA methylation in oogenesis occurs during the earliest stages of follicular development (53,54). Moreover, *DNMT3B* seems to be the major DNMT that ensures global DNA remethylation during blastocyst formation before implantation (54). Although the effect of maternal smoking during pregnancy on global and site-specific DNA methylation in the placenta and neonates has been well documented (55-58), its precise impact on DNA methylation and expression on *DNMT3B* in growing oocytes, as well as the long-term consequences on fetal growth and the timing of birth, is yet to be determined. Moreover, the implied associations between genetic polymorphisms and the tendency to smoke could be confounded by patient selection. However, studies on the association between smoking-related cancers and epigenomic alterations showed that cigarette smoke influenced *DNMT3B* gene expression, thus changing DNA methylation patterns (59-61).

Although our study was the first study conducted in women with SPTB, the association between *DNMT1* rs2162560, *DNMT3A* rs734693, *DNMT3B* rs2424913, and *DNMT3L* rs7354779 and birth outcome was evaluated in one previous study (27). In that study, only maternal *DNMT3B* rs2424913 minor allele was associated with an increased risk for PTB, confirming *DNMT3B* as a potential candidate gene for PTB. Furthermore, three independent studies found *DNMT3B* rs1569686 and rs2424913 to be maternal risk factors for Down syndrome (30,31,62), again confirming the importance of *DNMT3B* gene polymorphisms in human reproduction.

Although our study did not find an association between SPTB and the other tested polymorphisms in *DNMT1*, *DNMT3A*, and *DNMT3L* genes, they still represent good candidate genes for SPTB considering their functionality and the role DNMTs play in modifications during gametogenesis and pregnancy. Although *DNMT1* rs2228611 is located within exon 17 and is considered to be a synonymous mutation, according to *in silico* analysis it might affect splicing regulation (40). This polymorphism was also reported to affect LINE-1 methylation in women exposed to cadmium (63). *DNMT3A* rs1550117 is located 448 base pairs upstream of the transcription start site, and the A allele decreases its expression (64). Intronic *DNMT3L* rs2070565 is also a splice site variant (40). Additionally, there are other polymorphisms within these genes that should be considered for future analysis.

The potential limitations of our study include the analysis of only the maternal genotypes and the low number of patients in the subgroup analysis, which reduces the study power. Moreover, we did not adjust P value for multiple comparisons and multiple presented analyses. On the other hand, the strengths of our study include patient selection according to the standard clinical definition of SPTB, sufficient statistical power, and the use of peripheral blood samples for DNA analysis. Further genetic association and expression studies in different populations should evaluate the role of DNMT gene polymorphisms in SPTB.
